# Large isoform of MRJ (DNAJB6) reduces malignant activity of breast cancer

**DOI:** 10.1186/bcr1874

**Published:** 2008-03-07

**Authors:** Aparna Mitra, Rebecca A Fillmore, Brandon J Metge, Mathur Rajesh, Yaguang Xi, Judy King, Jingfang Ju, Lewis Pannell, Lalita A Shevde, Rajeev S Samant

**Affiliations:** 1 Department of Oncologic Sciences, Mitchell Cancer Institute, University of South Alabama, N University Blvd, Mobile, Alabama 36688, USA; 2 Department of Pharmacology and Department of Pathology, University of South Alabama, N University Blvd, Mobile, Alabama 36688, USA

## Abstract

**Introduction:**

Mammalian relative of DnaJ (MRJ [DNAJB6]), a novel member of the human DnaJ family, has two isoforms. The smaller isoform, MRJ(S), is studied mainly for its possible role in Huntington's disease. There are no reports of any biologic activity of the longer isoform, MRJ(L). We investigated whether this molecule plays any role in breast cancer. Our studies were prompted by interesting observations we made regarding the expression of MRJ in breast cancer cell lines and breast cancer tissue microarrays, as described below.

**Methods:**

Expression of MRJ(L) from several breast cancer cell lines was evaluated using real-time PCR. Relative levels of the small and large isoforms in breast cancer cell lines were studied using Western blot analysis. A breast cancer progression tissue microarray was probed using anti-MRJ antibody. MRJ(L) was ectopically expressed in two breast cancer cell lines. These cell lines were evaluated for their *in vitro *correlates of tumor aggressiveness, such as invasion, migration, and anchorage independence. The cell lines were also evaluated for *in vivo *tumor growth and metastasis. The secreted proteome of the MRJ(L) expressors was analyzed to elucidate the biochemical changes brought about by re-expression of MRJ(L).

**Results:**

We found that MRJ(L) is expressed at a significantly lower level in aggressive breast cancer cell lines compared with normal breast. Furthermore, in clinical cases of breast cancer expression of MRJ is lost as the grade of infiltrating ductal carcinoma advances. Importantly, MRJ staining is lost in those cases that also had lymph node metastasis. We report that MRJ(L) is a protein with a functional nuclear localization sequence. Expression of MRJ(L) via an exogenous promoter in breast cancer cell line MDA-MB-231 and in MDA-MB-435 (a cell line that metastasizes from the mammary fat pad) decreases their migration and invasion, reduces their motility, and significantly reduces orthotopic tumor growth in nude mice. Moreover, the secreted proteome of the MRJ(L)-expressing cells exhibited reduced levels of tumor progression and metastasis promoting secreted proteins, such as SPP1 (osteopontin), AZGP1 (zinc binding α_2_-glycoprotein 1), SPARC (osteonectin), NPM1 (nucleophosmin) and VGF (VGF nerve growth factor inducible). On the other hand, levels of the secreted metastasis-suppressor KiSS1 (melanoma metastasis suppressor) were increased in the secreted proteome of the MRJ(L)-expressing cells. We confirmed by quantitative RT-PCR analysis that the secreted profile reflected altered transcription of the respective genes.

**Conclusion:**

Collectively, our data indicate an important role for a totally uncharacterized isoform of DNAJB6 in breast cancer. We show that MRJ(L) is a nuclear protein that is lost in breast cancer, that regulates several key players in tumor formation and metastasis, and that is functionally able to retard tumor growth.

## Introduction

Mammalian relative of DnaJ (MRJ) is a class II DnaJ/heat shock protein (Hsp)40 family protein [1,2]. The human *MRJ *gene has been mapped to chromosome 7q36.3 and encodes two spliced variants (reported in GenBank) [3]: isoform a (referred to here as MRJ [L]) and isoform b (MRJ [S]). Murine MRJ has been shown to be essential for murine placental development [4]. MRJ(S) has also been implicated as an important chaperone in Huntington's disease [5,6]. Over-expressed MRJ(S) effectively suppressed polyglutamine-dependent protein aggregation, caspase activity, and cellular toxicity in neurons. MRJ(S) specifically has also been shown to regulate keratin-8/18 filament organization [7]. Interesting recent studies have shown a role for MRJ(S) in blocking calcineurin-induced cardiomyocyte hypertrophy [8].

All studies of MRJ have focused on the shorter isoform (isoform b, 242 amino acids). Our study elucidates the role of the longer isoform (isoform a, 326 amino acids) in breast cancer cells. Here, we report for the first time that MRJ(L) levels are almost undetectable in aggressive breast cancer cells and in advanced grade infiltrating ductal carcinoma. MRJ(L) localizes to the nucleus and suppresses tumorigenicity and metastasis of breast cancer cells. Furthermore, cells expressing MRJ(L) show an altered profile of the secreted proteome. Several secreted proteins that are known to play important roles in promoting cancer progression are downregulated. This is concomitant with upregulation of a breast and melanoma metastasis suppressor protein, namely KiSS1 (melanoma metastasis suppressor; metastin).

## Materials and methods

### Cell culture

MDA-MB-231 and MDA-MB-435 cells were maintained as described before [9]. Transfected cells were grown in presence of 500 μg/ml G418 (Invitrogen, Carlsbad, CA, USA). Cells of lines MCF10.DCIS.com, MCF10CAcl.a, and MCF10CAcl.d were cultured in Dulbecco's modified Eagle's medium (DMEM)/F12 with 5% horse serum, cholera toxin (100 ng/ml), insulin (10 μg/ml), hydrocortisone (500 ng/ml) and epidermal growth factor (EGF; 25 ng/ml). MCF7 was cultured in DMEM/F12 containing 5% horse serum and insulin (10 μg/ml). All cells were maintained at 37°C with 5% carbon dioxide in a humidified atmosphere.

MCF10DCIS.com (locally aggressive) and metastatic variants MCF10CAcl.a and MCF10CAcl.d are isogenic cell lines derived from *in vivo *passages of MCF10AT (tumorigenic) in nude mice [10,11]. These cell lines were obtained from the Barbara Ann Karmanos Cancer Institute (Detroit, MI, USA).

MDA-MB-435 was reported to be derived from the pleural metastases of a 46-year-old woman with breast carcinoma. It forms progressively growing tumors that form metastases in the lungs and regional lymph nodes after injection into the mammary fat pads of 5-week-old athymic nude mice [12]. The origin of MDA-MB-435 has been questioned because it expresses melanoma-associated genes and is reported to originate from the M14 melanoma line [13-15]. Its breast cancer origin was contested based on the fact that it expresses milk proteins [16,17]. It is also pertinent to note that MDA-MB-435 metastasizes from the mammary fat pad but rarely from the subcutaneous site. In the present study this cell line was used because it metastasizes from the mammary fat pad.

### Plasmids and cell lines

MRJ(L) cDNA was subcloned into mammalian expression vector pIRES2-EGFP (Clontech, Mountain View, CA, USA) to obtain pIRES2-EGFP-MRJ(L). MDA-MB-231 and MDA-MB-435 cells were transfected with pIRES2-EGFP-MRJ(L). We used the Lipofectamine 2000 reagent (Invitrogen). Briefly, 2 × 10^6 ^cells were seeded in a 60 mm dish 1 day before transfection. Plasmid (8 to 10 μg) was mixed the Lipofectamine 2000 reagent, as recommended by the manufacturer. The DNA-Lipofectamine 2000 complex (final volume 1 ml) was added to the plate containing 4 ml serum-free DMEM/F12. Twelve hours later the media were replaced with growth medium without any selection. Transfection was monitored by examining the tranfectants for the green fluorescence. Cells were detached approximately 45 hours after transfection. Using FACSVantage SE (Becton Dickinson), the top 20% fluorescent (enhanced green fluorescent protein [EGFP]) cells were selected (correspondingly high MRJ [L] expressors because of the internal ribosome entry site [IRES]). The expressor cells were passaged in medium with G418. We used the expressors as a pooled population to avoid any clonal variation. All cell lines were used within 10 to 12 passages to avoid variations.

MRJ(L) with wild-type and mutant nuclear localization signal (NLS) were cloned in frame with a carboxyl-terminal EGFP tag in the vector pEGFP-N1. The wild-type NLS from amino acids 305 to 313 (KRKKQKQR) was mutated to (RPDRPETT) using PCR. The corresponding plasmids are called as pEGFP-N1-MRJ(L)^WT ^and pEGFP-N1-MRJ(L)^mut^.

### Collection of Serum free media and Western blot for secreted proteins

Cells (2 × 10^6^) were plated in 10 cm plate containing appropriate growth medium overnight. The growth medium was replaced with 3 ml serum-free phenol-free DMEM/F12. The conditioned medium was harvested 18 hours later, spun at 1,300 rpm at 4°C for 10 minutes, and concentrated eightfold using a Millipore Amicon Ultra 4 Centrifugal Filter Unit (Billerica, MA, USA) with a 10 KDa cut-off. The concentrated conditioned media samples were resolved on SDS-PAGE and immunoblotted for proteins of interest.

### Immunoblotting

Plates (10 cm) at 90% confluence were washed twice with calcium and magnesium-free phosphate buffered saline (PBS) and lysed with cold lysis buffer (150 mmol/l NaCl, 50 mmol/l Tris, 1% NP-40, and protease and phosphatase inhibitors). The lysates were kept on ice for 1 hour and centrifuged at 10,000 rpm for 30 minutes at 4°C. Protein concentration was measured using Bradford reagent (BioRad Laboratories, Hercules, CA, USA). Proteins (20 μg) were subjected to SDS-PAGE and transferred to polyvinylidene fluoride membrane (0.2 μm). The membranes were blocked with 5% skimmed milk in Tris-buffered saline Tween-20 (1 mol/l Tris [pH 7.5], 9% NaCl and 0.05% Tween-20) and incubated with primary antibodies overnight at 4°C. After washes in Tris-buffered saline Tween-20 and incubation with the respective horseradish peroxidase-tagged secondary antibody, the blots were developed using SuperSignal™ (Pierce, Rockford, IL, USA). For detection of MRJ, 5% milk in PBS containing 0.2% Tween-20 was used, and for detection of osteopontin, 3% bovine serum albumin in PBS containing 0.05% Tween was used as blocking buffer.

We used the following antibodies (dilutions given in parenthesis): anti-DNAJB6 antibody (1:5,000) from Abnova Corporation (Taipei City, Taiwan); anti-osteopontin (1:2,500) from Sigma (St. Louis, MO, USA); anti-KiSS1 antibody (1:500) from Abcam (Cambridge, MA, USA); anti-osteonectin (1:1,000) from Haematologic Technologies Inc. (Essex Junction, VT, USA); anti-nucleophosmin (1:500) from Abcam; anti-zinc α_2_-glycoprotein (1:500); anti-VGF nerve growth factor (1:500) from Santa Cruz Biotechnology Inc. (Santa Cruz, CA, USA); and β-actin (1:30,000) from BioRad.

### Wound healing assay

Cells were cultured to confluence on six-well plates, which were previously externally marked with parallel lines to use as guides for the subsequent photography. A central linear wound (perpendicular to the guide lines) was made with a 200 μl sterile pipet tip. Media were changed gently to remove any floating cells. Phase micrographs of the wound cultures were taken at 0 and 16 hours (average doubling time is 18 to 20 hours). The photographs were analyzed by measuring the distance from the wound edge of the cell sheet to the original wound site. Migration activity was calculated as the mean distance between edges in 12 fields per well. Each test group was assayed in triplicate, and the results are expressed relative to vector control cell migration.

### Migration and invasion assay

Migration and invasion assays were conducted using 8 μm polyethylene terpthalate filters (BD Pharmingen, San Diego, CA, USA), as described previously [18]. Cells migrated to the lower sides of the trans-well were stained using Diff-Quik^® ^reagent (Diff Quik is from International Medical Equipment Inc., San Marcos, CA, USA) and the cell number was counted under a microscope. Each test group was assayed in triplicate. Four different fields of each insert were photographed at 10× magnification using a Zeiss Axiocam 200 M microscope (Zeiss Axiocam : Carl Zeiss Microimaging Inc. Thornwood, NY, USA.). Each field was divided into quadrants, and cells in diagonally opposite quadrants were counted.

### Soft agar colonization assay

Cells (2 × 10^3^) suspended in 0.35% agar were plated onto a layer of 0.75% bactoagar in DMEM/F12 (5% fetal bovine serum) in six-well tissue culture dishes. Visible colonies (> 50 cells) were counted after 15 days with the aid of a dissecting microscope [9].

### Nuclear isolation

Nuclear protein extraction was performed as described previously [19]. Briefly, a confluent monolayer of cells was washed twice in ice-cold PBS. Buffer A (10 mmol/l Hepes [pH 7.9], 10 mmol/l KCl, 0.1 mol/l EDTA, 200 μl of 10% IGEPAL, 1 mmol/l DTT, and protease inhibitor cocktail) was added to the monolayer and the plate was maintained at room temperature for 10 minutes. The lysate was spun at 15,000 *g *for 3 minutes at 4°C. The supernatant was saved as cytosolic fraction. The pellet was suspended in buffer B (20 mmol/l Hepes [pH 7.9], 0.4 mol/l NaCl, 1 mmol/l EDTA, 10% glycerol, 1 mmol/l DTT, and protease inhibitor cocktail) and mixed vigorously for 2 hours at 4°C. The nuclear lysate was obtained by centrifuging at 15,000 *g *for 5 minutes at 4°C.

### Confocal microscopy

COS7 or MDA-MB-231 cells were transfected with pEGFP-N1-MRJ(L)^WT ^or pEGFP-N1-MRJ(L)^mut ^using Lipofectamine 2000 (Invitrogen), in accordance with the manufacturer's instructions. Cells were visualized 32 hours after transfection using Leica Microsystems TCS SP2 confocal microscope with 63× water immersion objective (Leica Microsystems Inc.Bannockburn, IL USA). Leica Confocal Software version 2.61 was used for data analysis and fluorescence/differential interference contrast image overlays.

### Tumor growth and metastasis assays

Cells (10^7 ^cells/ml) were re-suspended in ice-cold Hanks balanced salt solution and 0.1 ml was injected into exposed axillary mammary fat pads of anesthetized (ketamine 80 mg/kg, xylazine 14 mg/kg), 6-week-old, female athymic mice (Harlan Sprague-Dawley, Indianapolis, IN, USA). Tumor size was measured weekly and mean tumor diameter was calculated as previously reported [9]. The tumor growth was followed for a period of 4 weeks.

The experimental metastasis assay was performed by injecting 2.5 × 10^5 ^MDA-MB-231 cells (in 0.2 ml Hanks balanced salt solution) into the lateral tail vein of female athymic mice aged 3 to 4 weeks, as previously described [9]. After a period of 6 weeks, mice were killed; lungs were removed, rinsed with PBS, and fixed in diluted Bouin's solution (20% Bouin's fixative in neutral buffered formalin) before quantification of surface metastasis. For all of the experiments into tumor growth and metastasis, eight mice were used per group and each experiment was repeated once. Animals were maintained under the guidelines of the National Institutes of Health and Institutional Animal Care and Use Committee (IACUC) of the University of South Alabama, Mobile. Food and water were provided *ad libitum*.

### Secreted proteome analysis

The analysis of secreted proteome was conducted as previously described [20]. Briefly, proteins from the conditioned serum-free medium were concentrated using a tC2 reversed-phase Sorbent column. The protein sample in 0.1% trifluoroacetic acid was loaded onto the cartridge and washed with 0.1% trifluoroacetic acid. Proteins were eluted with increasing amounts of acetonitrile in 0.1% trifluoroacetic acid from 30% to 70% at 0.1 ml/minute, concentrated to dryness, and digested with trypsin. The resulting peptides were analyzed by liquid chromatography-mass spectrometry. Electrospray tandem mass spectrometry was performed with a Q-TOF Ultima API-US mass spectrometer (Waters, Milford, MA, USA) equipped with a nanoflow electrospray.

The resulting data files were searched using an in-house MASCOT search engine (version 2.1.03; Matrix Science Ltd, London, UK) [21]. Ion scores higher than 35 (*P *< 0.05) were considered significant. Only proteins matching at least two peptides in MASCOT were accepted.

### RNA isolation and cDNA synthesis and real-time quantitative RT-PCR

TRIzol reagent (Invitrogen) was used to isolate total RNA from cultured cells. RNA was treated with DNase I (Promega, Madison, WI, USA). cDNA synthesis was carried out using a cDNA synthesis kit (Applied Biosystems Inc., Foster City, CA, USA) using 1 μg total RNA as the template and random primers. Normal breast RNA was purchased from Clontech. Real-time quantitative RT-PCR analysis was performed on the experimental mRNAs. The PCR primers and probes for KiSS1, nucleophosmin (NPM1), osteonectin (SPARC), osteopontin (SPP1), zinc binding α_2_-glycoprotein 1 (AZGP1) and VGF nerve growth factor inducible (VGF), and endorse control gene glyceraldehyde 3-phosphate dehydrogenase (GAPDH) were purchased from Applied Biosystems Inc. Quantative RT-PCR was performed on an ABI 7500 HT instrument (Applied Biosystems, Foster City, CA, USA). The gene expression Δ CT values of mRNAs from each sample were calculated by normalizing with internal control GAPDH and relative quantitation values were plotted using SDS software v1.2 (Applied Biosystems Inc.). Real-time quantitative RT-PCR analysis was performed on the experimental mRNAs in triplicate, and the experiment was repeated once from an independent passage to confirm the findings.

### Tissue array analysis

Breast carcinoma progression array (CC08-00-001) developed by Cybrdi Inc. (Frederick, MA, USA) was probed by Cybrdi Inc. The arrays were stained by 1:100 dilution (10 μg/ml) of DNAJB6 monoclonal antibody. Mouse IgG_1 _from Vector PK-6102 (Vector Laboratories, Burlingame, CA, USA) was used for the isotype background control. Photomicrographs were taken in the area of most intense and diffuse staining for MRJ. The intensity of staining of tumor cells was assessed as 0 (no staining) to 3 (strongest possible intensity of staining). The immunoscore was derived as the product of the percentage of cells at each intensity and the corresponding intensity [18]. The products were added to obtain an average immunoscore for the group.

### Statistical analysis

Statistical differences between groups were assessed using the *t*-test or analysis of variance, using GraphPad Prism 4 statistical software (San Diego, CA, USA).

### Ethics approval

The animal experiments were performed under protocol #06042, approved by the IACUC committee of the University of South Alabama, Mobile.

## Results

### MRJ(L) is expressed at a significantly lower level in breast cancer cell lines

**Figure 1:**
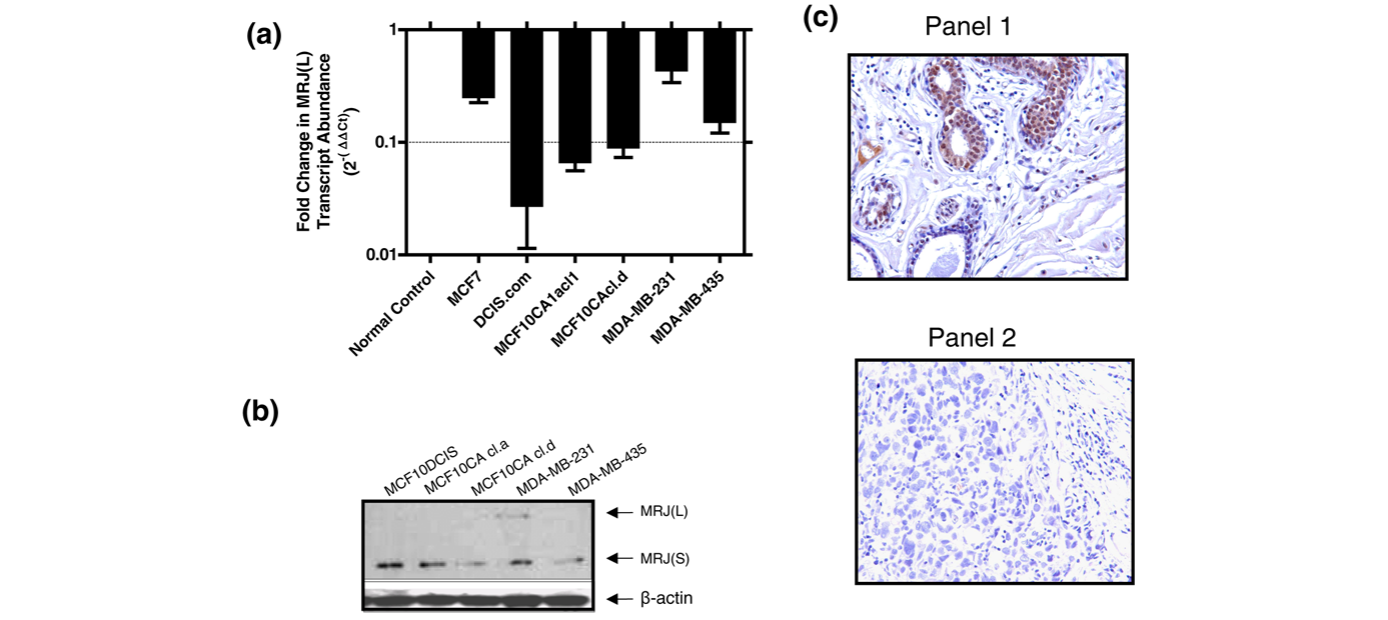
Expression of MRJ in breast cancer cell lines and tissues. **(a) **Expression of mammalian relative of DnaJ (MRJ) long isoform (MRJ [L]) is significantly lower in various breast cancer cell lines as compared with that observed in RNA from normal breast. Real-time quantitative RT-PCR was used to assess expression of MRJ(L) relative to endorse control gene glyceraldehyde 3-phosphate dehydrogenase (GAPDH). The data are represented as fold change in the abundance of the MRJ(L) transcripts using commercially available normal breast RNA as a calibrator. Each reaction was carried out in triplicate, and the experiment was repeated once with RNA from the same cell lines at a different passage. The error bars represent the standard error. **(b) **Expression of MRJ isoforms in various breast cancer cell lines. An equal amount of protein lysate (20 μg) was resolved on SDS-PAGE and immunoblotted for levels of MRJ isoforms. Equal loading was confirmed by comparable β-actin signal. **(c) **Tissue microarray staining for MRJ. A breast carcinoma progression array (CC08-00-001; developed by Cybrdi Inc.) was stained by 1:100 dilution (10 μg/ml) of DNAJB6 monoclonal antibody. Mouse IgG_1 _was used for the isotype background control. Photomicrographs were taken in the area of most intense and diffuse staining for MRJ. The photomicrographs are representative images showing staining patterns of MRJ. Panel 1 corresponds to cystic hyperplasia, and panel 2 corresponds to infiltrating ductal carcinoma grade III.

We assessed the expression of MRJ(L) in breast cancer cell lines using a real-time quatitative RT-PCR. As seen in Figure 1a, all breast cancer cell lines tested by us express very low levels of MRJ(L) compared with the expression seen in normal breast. However, among the cancer cell lines, MDA-MB-231 expressed MRJ(L) at a relatively high level.

We validated this observation at the protein level by an immunoblot for MRJ. As seen in Figure 1b, all cell lines exhibited a clearly detectable level of MRJ(S) (28 kDa). Corresponding to the quatitative RT-PCR data, the larger isoform MRJ(L) (38.6 kDa) was not detectable. Upon prolonged exposure, MDA-MB-231 cells exhibited weakly detectable MRJ(L).

### The expression of MRJ is lost in advanced breast cancer

We probed a breast carcinoma progression tissue microarray using antibody specific to MRJ. The antibodies currently available are unable to distinguish between small and large isoforms. The results showed that 80% of the normal breast cases (four out of five) were positive for MRJ staining. We also found that among benign cases nearly half of the tissues stained positive (48%), whereas in infiltrating ductal carcinoma (IDC) cases (grades I and II) 50% cases stained positive (six out of 12 cases). Importantly, only one out of 6 cases (17%) of IDC grade III stained positive, whereas all six cases of IDC with lymph node metastasis exhibited complete absence of staining. Table 1 also outlines the average immunoscores, which decrease with aggressiveness of the tumor. Figure 1c shows intense nuclear and cytoplasmic staining in cystic hyperplasia (panel 1). In contrast, this staining is absent in IDC grade III (panel 2).

**Table 1:** Immunostaining of MRJ is decreased in aggressive breast cancer specimen

Tissue type	Total assessed	Stained positive	Percent positive	IHC Scores
Normal	5	4	80	1.6
Benign	25	12	48	0.54
IDC stage I and II	12	6	50	0.45
IDC stage III	6	1	17	0.108
IDC with lymph node metastasis	6	0	0	0

A breast carcinoma progression array (CC08-00-001; developed by Cybrdi Inc.) was stained by 1:100 dilution (10 ug/ml) of DNAJB6 monoclonal antibody. Mouse IgG_1 _was used for the isotype background control. Photomicrographs were taken in the area of most intense and diffuse staining for mammalian relative of DnaJ (MRJ). The intensity of staining of tumor cells was assessed as 0 (no staining) to 3 (strongest possible intensity of staining). The immunoscore was derived as the product of the percentage of cells at each intensity and the corresponding intensity [18]. The products were added to obtain an average immunoscore for the group. IDC, infiltrating ductal carcinoma; IHC, immunohistochemistry.

### There are two distinct isoforms of MRJ

**Figure 2:**
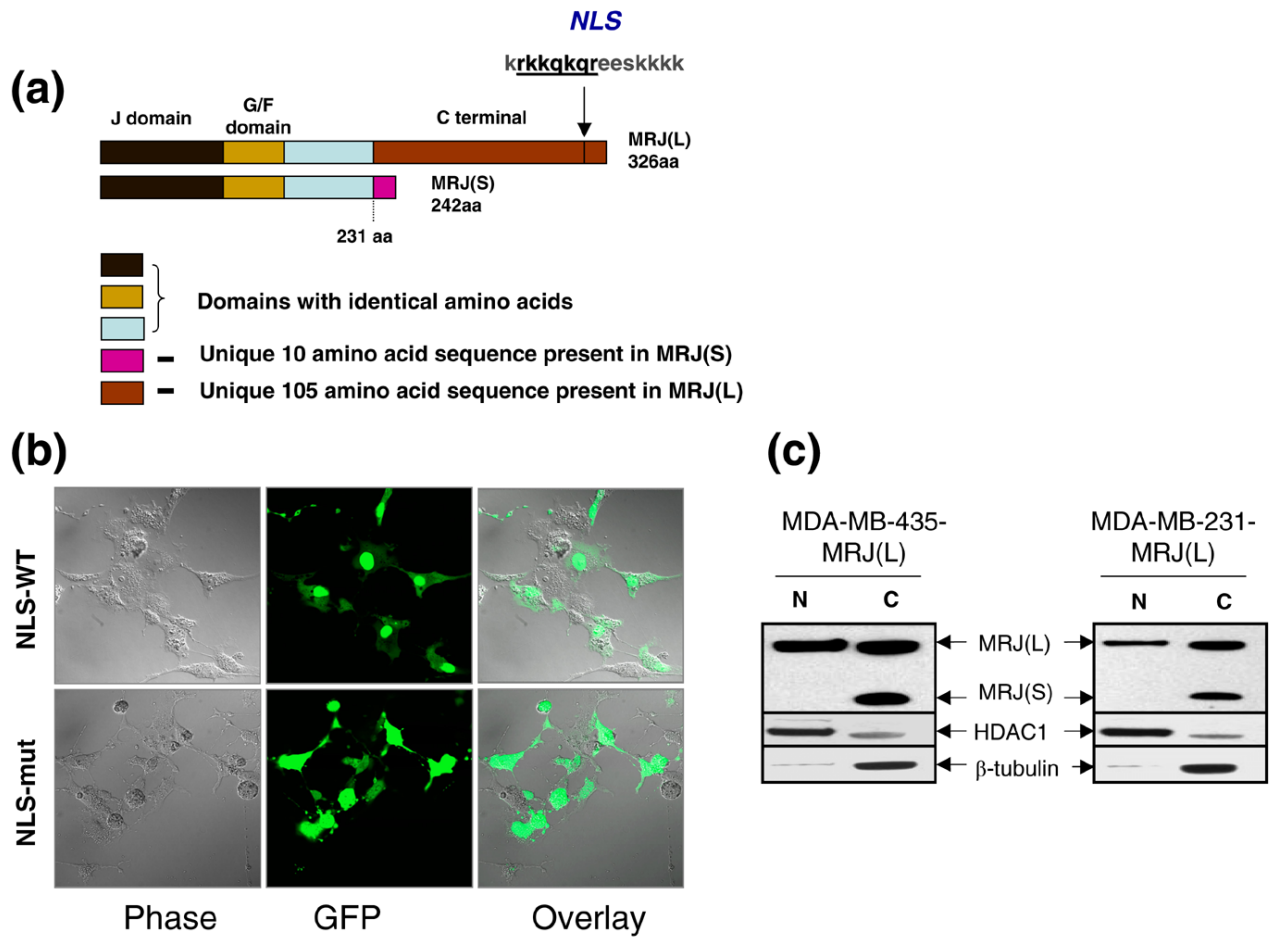
MRJ(L) localizes to nucleus. **(a) **Comparison of the isoforms of DNAJB6 (mammalian relative of DnaJ [MRJ]). Various domains are indicated with distinct colors. Domains common to both DNAJB6 isoforms bear the same color code. Domains specific to each isoform bear unique color code. The nuclear localization signal (NLS) sequence is represented using single letter code for the amino acids. The amino acids underlined and in bold represent the strongest NLS. **(b) **COS7 cells were transfected with fusion protein pEGFP-N1-MRJ(L)^WT ^or pEGFP-N1-MRJ(L)^mut^. Cells were visualized 32 hours after transfection using a confocal microscope with 63× water immersion objective. MRJ(L)^WT^-EGFP was predominantly in the nucleus, whereas the mutant NLS protein, MRJ(L)^mut^-EGFP, exhibits a uniform distribution throughout the cell. **(c) **Equal protein from nuclear (N) and cytoplasmic (C) extracts (20 μg) from MRJ long isoform (MRJ [L]) expressors of MDA-MB-435 and MDA-MB-231 were analyzed for the presence of MRJ(L). Histone deacetylase 1 (HDAC1) was used as a marker to validate the purity of the nuclear fraction. β-Tubulin was used as cytoplasmic marker. MRJ(L) migrates at the apparent molecular weight of 38 kDa, MRJ short isoform (MRJ [S]) at 28 kDa, and HDAC1 at 55 kDa. EGFP, enhanced green fluorescent protein.

The full length DNAJB6 isoform a (MRJ [L]) is comprised of 326 amino acids, whereas the shorter isoform b, (MRJ(S)), has 242 amino acids. MRJ(S) lacks the carboxyl-terminal 95 amino acids of MRJ(L) but contains an additional 10 amino acids (KEQLLRLDNK). Apart from the differences at the carboxyl-terminus, both isoforms share identical structure, containing a conserved J domain (70 amino acids) and a glycine/phenylalanine domain (Figure 2a) [22].

### MRJ(L) has a functional nuclear localization signal

PSORT II software (PSORT II is based on the collaboration of Kenta Nakai, Ph.D.(Human Genome Center, Institute for Medical Science, University ot Tokyo, Japan), with Paul Horton (National Institute of Advanced Industrial Science and Tachnology, Tokyo, Japan.,) [23] predicted the presence of an NLS in MRJ(L) from amino acids 305 to 320 (KRKKQKQREESKKKK). We constructed an in-frame fusion of the NLS using requisite primers and PCR, and generated a mutant NLS by inserting point mutations. The mutated NLS sequence is RPDRPETTEESKKKK. Both the wild-type and mutated NLSs were cloned into the pEGFP-N1 plasmid to generate an in-frame EGFP fusion. Upon transfection of COS7 cells with these constructs, we see that the wild-type fusion protein MRJ(L)^WT^-EGFP is predominantly localized to the nucleus, whereas the mutant NLS protein MRJ(L)^mut^-EGFP exhibits a uniform distribution throughout the cell (Figure 2b). Similar results were observed when the MRJ(L)^WT^-EGFP and the MRJ(L)^mut^-EGFP fusion proteins were tested in breast cancer cell line MDA-MB-231. The analysis of nuclear and cytoplasmic protein fractions obtained from cells ectopically expressing MRJ(L) (stable transfectants are described in the following section) also reveals nuclear as well as cytoplasmic localization of MRJ(L) in MDA-MB-231 and MDA-MB-435 cells. However, MRJ(S) was found to be almost exclusively cytoplasmic (Figure 2c).

### Cell lines ectopically expressing stable MRJ(L) were established

**Figure 3:**
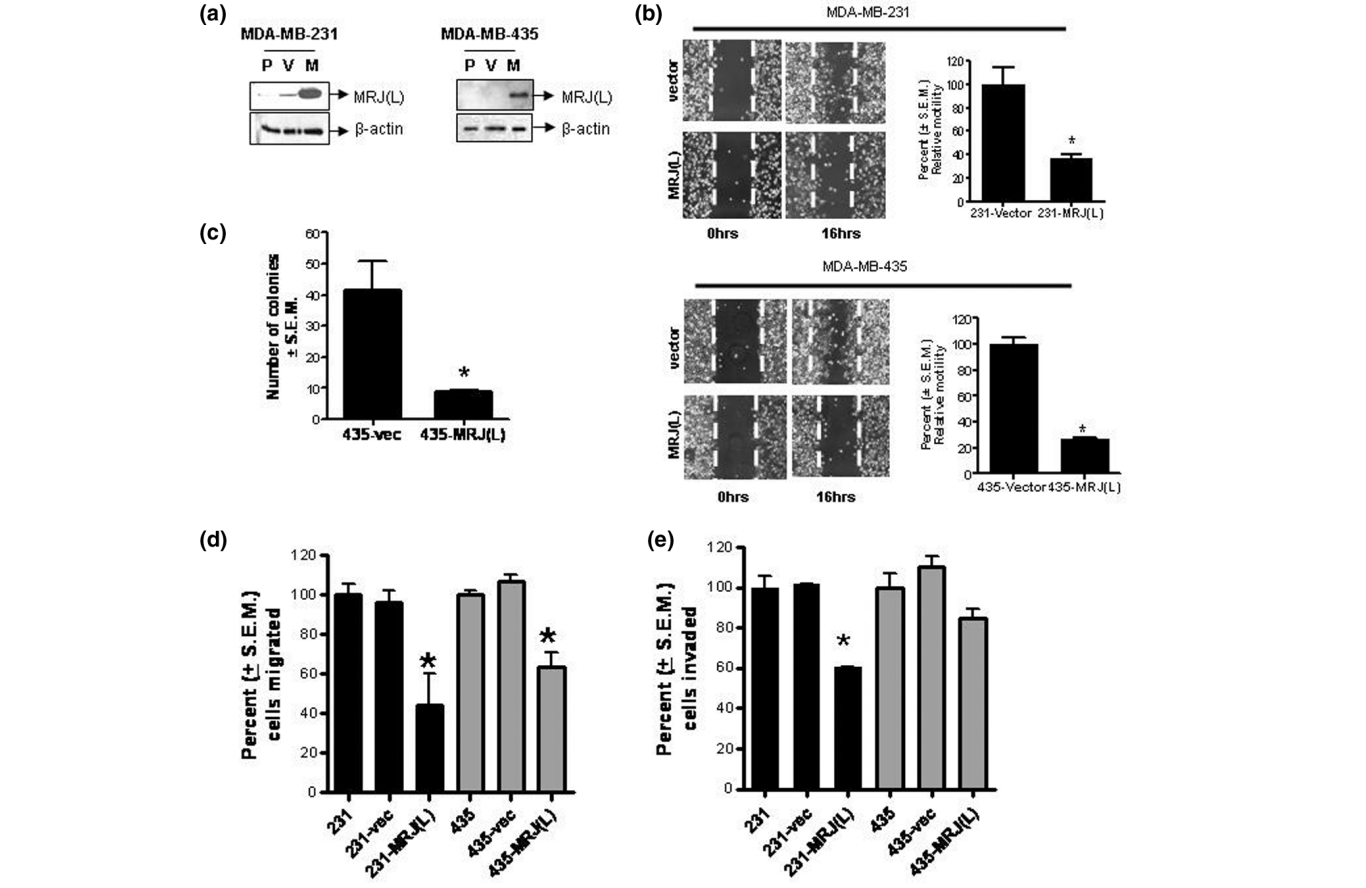
Analysis of *in vitro *attributes of tumor progression. **(a) **Equal amounts (20 μg) of protein extracts from mammalian relative of DnaJ (MRJ) long isoform (MRJ [L]) expressors (M) of MDA-MB-231 and MDA-MB-435 were compared with the parent (P) and vector control (V) for the level of MRJ(L). MRJ(L) migrates at the apparent molecular weight of 38 kDa. Equal loading was confirmed by comparable β-actin signal. **(b) **MDA-MB-231-MRJ(L), referred to as 231-MRJ(L), and MDA-MB-435-MRJ(L), referred to as 435-MRJ(L), exhibited decreased migration compared with the corresponding vector controls (231-vec and 435-vec) in wound healing assay. Cells were cultured to confluence on premarked six-well plates. A central linear wound was made with a 200 μl sterile pipet tip. Phase micrographs of the wound cultures were taken at 0 and 16 hours. The photographs were analyzed by measuring the distance from the wound edge of the cell sheet to the original wound site. The dotted white lines in the photomicrographs indicate the original position of the wound. Migration activity was calculated as the mean of the distance between the edges in 12 independent fields per well. Each test group was assayed in triplicate, and the results are expressed relative to vector control cell migration. *P < 0.05. **(c) **MDA-MB-435-MRJ(L) exhibited reduced anchorage-independent growth compared with vector control. Cells (2 × 10^3^) suspended in 0.35% agar were plated onto a layer of 0.75% bactoagar in Dulbecco's modified Eagle's medium/F12 (5% fetal bovine serum) in six-well tissue culture dishes. Visible colonies (> 50 cells) were counted after 15 days with the aid of a dissecting microscope. The results are expressed as mean number of colonies ± standard error of the mean. *P < 0.05. **(d,e) **MRJ(L) expressors of MDA-MB-231 and MDA-MB-435 exhibit significantly reduced ability to migrate through transwell (panel d) and are retarded in terms of their ability to invade through Matrigel™- coated filters (panel e). Migration and invasion assays were conducted using 8 μm polyethylene terphthalate filters, as previously described [18]. Cells migrated to the lower sides of the transwell were stained using Diff-Quik^® ^reagent and the cells were counted under a microscope. Each test group was assayed in triplicate. Four different fields of each insert were photographed; each field was divided into quadrants and cells in diagonally opposite quadrants were counted.

MDA-MB-435 and MDA-MB-231 cells were stably transfected with MRJ(L)-pIRES2-EGFP as well as with the empty vector. The transfectants were subjected to fluorescence-activated cell sorting to collect cells with greater fluorescence intensity (top 20%). Because of the presence of an IRES, the high fluorescence corresponds to high level of expression of MRJ(L). This population shorted via fluorescence-activated cell sorting was expanded by propagation using G418 resistance and used for subsequent experiments. The expression of MRJ(L) was confirmed by Western blot analysis (Figure 3a).

### Analysis of *in vitro *attributes of tumor progression

The *in vitro *attributes of tumor progression were studied using wound healing assay, invasion, migration and soft agar colonization. MRJ(L) expressors of MDA-MB-231 exhibited 50% motility in a wound healing assay compared with the vector control (Figure 3b). The trend was same for MRJ(L) expressors of MDA-MB-435 MRJ(L), which exhibited 30% motility (Figure 3b). Expressors of MDA-MB-435 showed a highly reduced (10% of vector control) capacity for anchorage-independent growth when tested by colony formation ability in soft agar. Moreover, the colonies that formed in MDA-MB-435-MRJ(L) were small and grew much slower than did the vector control (Figure 3c). The MDA-MB-231 cells did not exhibit a significant change in the soft agar colonization, although the overall trend appeared to be lower than vector control (data not shown). Additionally MRJ(L) expressors of MDA-MB-231 exhibited reduced capacity to migrate (40% of the vector control) and invade (60% of the vector control) through Matrigel™ (BD biosciences San Jose, CA, USA) (Figure 3d, e). The MRJ(L) expressors of MDA-MB-435 also exhibited reduced migration (60% of the vector control) but showed a modest decrease in invasion (85% of the vector control; Figure 3d, e).

### MRJ(L) retards tumor growth and reduces lung colonization

**Figure 4:**
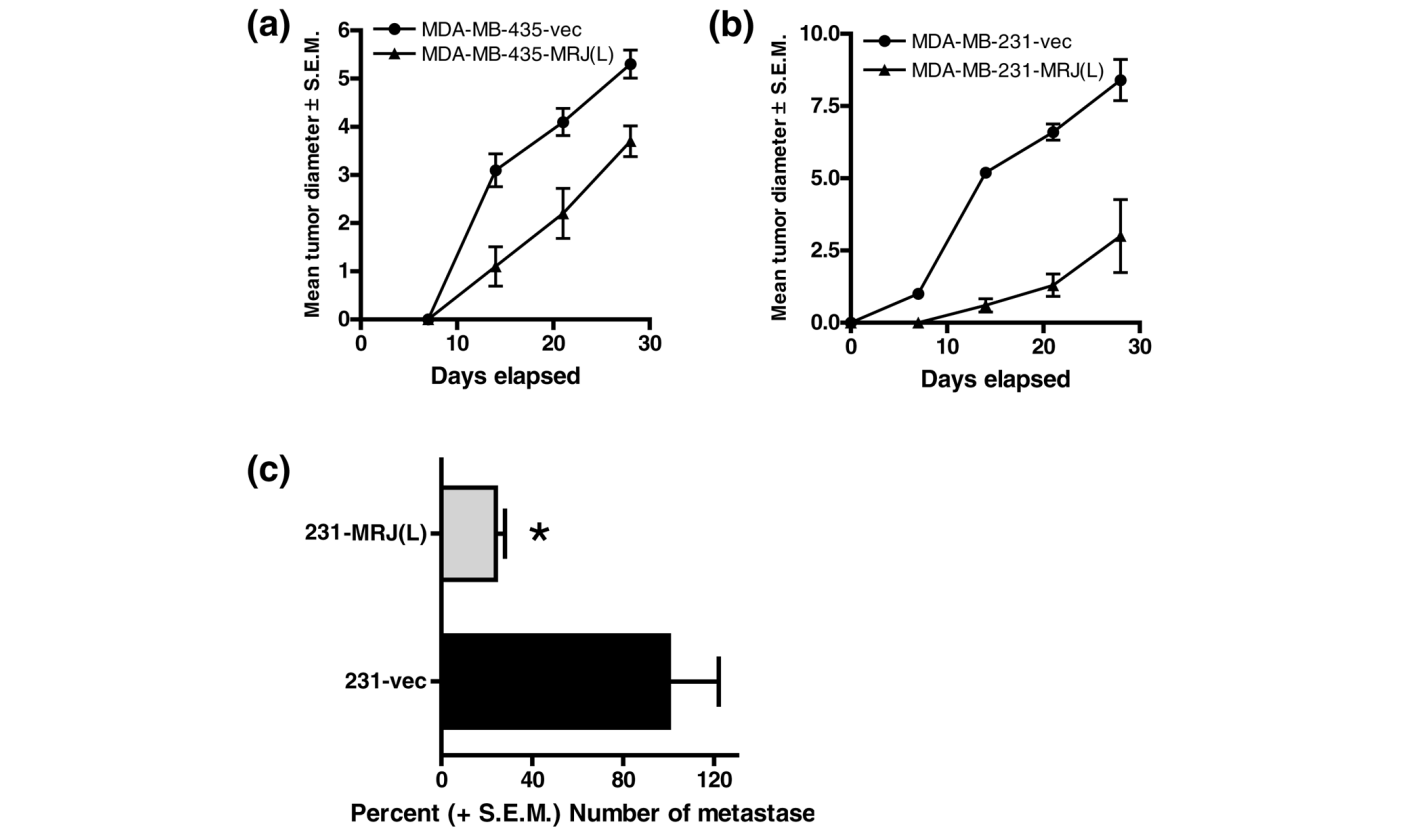
MRJ(L) retards tumor growth rate and metastasis. **(a,b) **Mammalian relative of DnaJ (MRJ) long isoform (MRJ [L]) expressors and corresponding vector control cells (10^6^/site) from MDA-MB-435 (panel a) and MDA-MB-231 (penal b) were injected into exposed axillary mammary fat pads of 6-week-old, female athymic mice. Tumor size was measured weekly. Eight mice were used per group and the experiment was repeated once. The results are expressed as the mean tumor diameters ± standard error. **(c) **The experimental metastasis assay (lung colonization asay) was performed by injecting 2.5 × 10^5 ^MDA-MB-231 cells (in 0.2 ml) into the lateral tail vein of 3- to 4-week-old, female athymic mice. After a period of 6 weeks lungs were removed and fixed with 20% Bouin's fixative in neutral buffered formalin before quantification of surface metastasis. Eight mice were used per group and the experiment was repeated once.

The MRJ(L) expressing MDA-MB-231 and MDA-MB-435 and the corresponding empty vector transfectants were independently assayed for orthotopic (mammary fat pad) tumor growth in nude mice. The tumor growth of MRJ(L) expressors of both the cell lines were found to be retarded when compared with the empty vector control (Figure 4a, b).

The MRJ(L) expressing MDA-MB-231 cells exhibited a significantly decreased (*P *< 0.05) ability to establish pulmonary metastases upon tail vein injection into athymic mice. The MRJ(L) expressing cells showed 65% fewer lung metastases compared with the vector control cells (Figure 4d).

### The secreted proteome of the MRJ(L) expressors is altered

**Figure 5:**
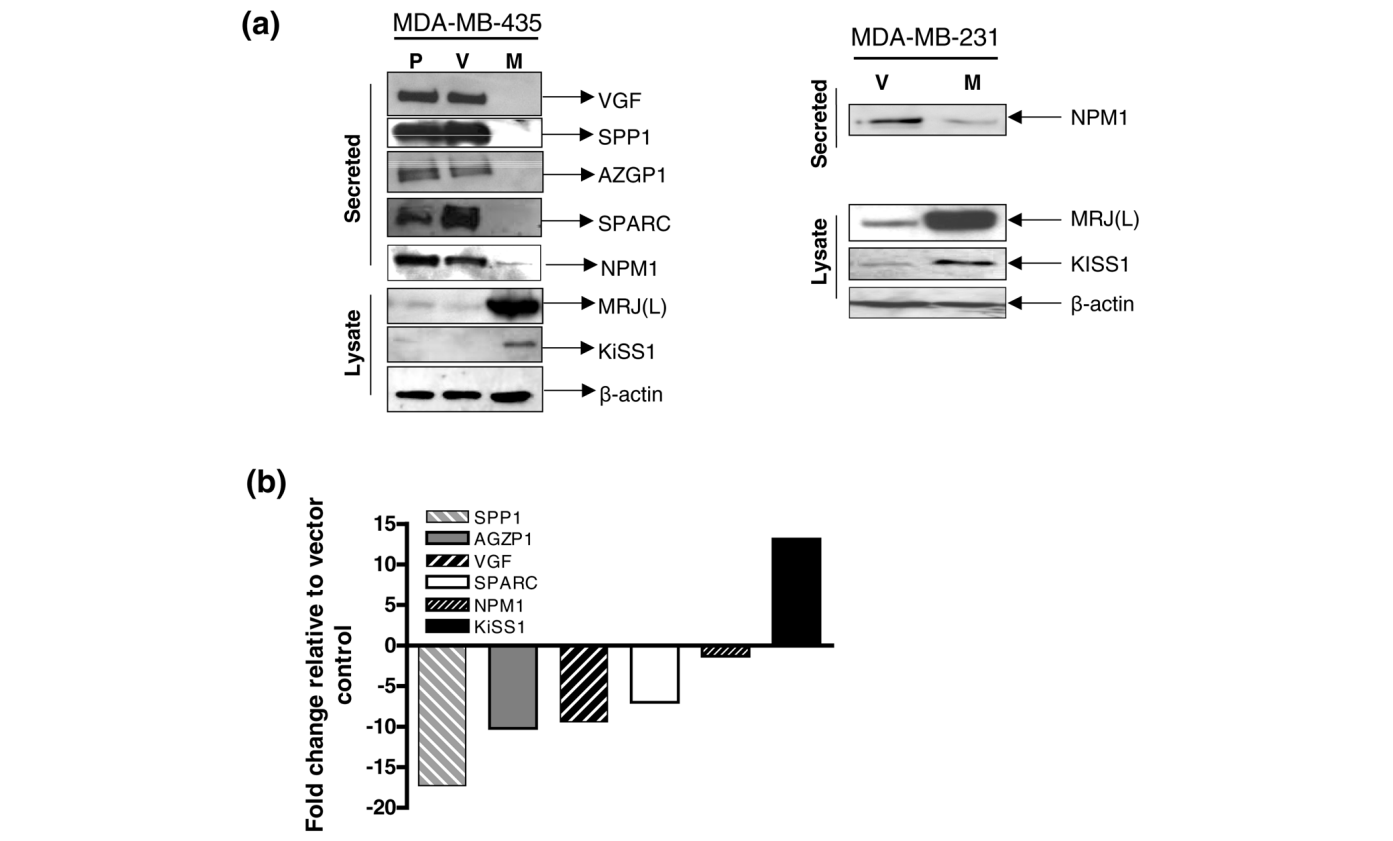
Confirmation of changes in level of secreted proteins. **(a) **The changes in the secreted proteome of mammalian relative of DnaJ (MRJ) long isoform (MRJ [L]) expressor observed by mass spectrometry were confirmed by Western blot analysis. Serum-free medium from equal number of MDA-MB-435 parent (P), vector (V), MRJ(L) expressors (M) or MDA-MB-231 vector (V), and MRJ(L) expressors (M) was probed for presence of osteopontin (SPP1), osteonectin (SPARC), VGF nerve growth factor inducible (VGF), and zinc binding α_2_-glycoprotein 1 (AZGP1). Equal amount of total protein extract (20 μg) of the same cells was probed for the level of KiSS1 (melanoma metastasis suppressor); simultaneously, the expression of MRJ(L) was confirmed. β-Actin was used to verify equal loading of the lysate. Apparent molecular weights of the proteins detected are given in parenthesis: VGF (90 kDa), SPP1 (62 kDa), AZGP1 (47 kDa), SPARC (40 kDa), nucleophosmin (NPM1; 37 kDa), MRJ(L) (38 kDa), and KiSS1 (15 kDa). **(b) **Real-time quantitative RT-PCR analysis was performed using RNA from the MDA-MB-435-vector and MDA-MB-435-MRJ(L) expressor. The PCR primers and probes for KiSS1, NPM1, AZGP1, SPARC, SPP1, and VGF and endorse control gene glyceraldehyde 3-phosphate dehydrogenase (GAPDH) were used. The reaction was performed for up to 40 cycles in triplicate. The gene expression Δ CT values of mRNAs from each sample were calculated by normalizing with internal control GAPDH. The fold change is represented as 2^-ΔΔCT^. Real-time quantitative RT-PCR analysis was performed on the experimental mRNAs in triplicate and the experiment was repeated once from an independent passage.

Conditioned cell-free and serum-free media from MRJ(L) expressing MDA-MB-435 were compared with the corresponding vector control. Our results (Table 2) show that SPP1, SPARC, AZGP1, VGF, and NPM1 were notably downregulated in MRJ(L) expressors. Conversely, we also found that the metastasis suppressor KiSS1 was upregulated in the secreted proteome of MRJ(L)-expressing cells compared with controls. Analysis of the serum-free medium by immunoblot analysis confirmed that levels of SPP1, SPARC, VGF, NPM1, and AZGP1 were below the limit of detection in MDA-MB-435-MRJ(L) culture medium (Figure 5a). The increased expression of KiSS1 in MRJ(L) expressors was confirmed by analyzing the cell lysate (Figure 5a). We found that MDA-MB-231 does not secrete SPP1 [24] and SPARC [25], which is in agreement with previously reported findings. Also, it does not secrete VGF and AZGP (data not shown). However, Western blot analysis revealed increased level of KiSS1 expression and decreased level of NPM1 in MRJ(L) expressing MDA-MB-231.

**Table 2:** Alteration in the secreted proteome of MRJ(L) expressor of MDA-MB-435

Protein name	NCBInr gi#	MDA-MB-435		MDA-MB-435 vec		MDA-MB-435-MRJ(L)	
		
		MASCOT position	Sequences matched	MASCOT position	Sequences matched	MASCOT position	Sequences matched
VGF (VGF nerve growth factor)	17,136,078	1	16	2	21	-	-
SPP1 (secreted phosphoprotein 1 [osteopontin])	4,759,166	5	5	7	7	-	-
AZGP1 (zinc α_2_-glycoprotein 1)	4,502,337	43	1	32	1	-	-
SPARC (secreted protein, acidic, cysteine rich [osteonectin])	4,507,171	84	2	36	3	-	-
NPM1 (nucleophosmin 1)	10,835,063	22	3	46	1	-	-
KiSS1 (melanoma metastasis suppressor)	29,571,104	-	-	-	-	14	2

The analysis of secreted proteome was carried out using secreted proteome from MDA-MB-435. Proteins from the conditioned serum-free medium were concentrated using a tC2 reversed-phase sorbent column and digested with trypsin. The resulting peptides were analyzed by electrospray tandem mass spectrometry using a Q-TOF Ultima API-US mass spectrometer equipped with a nanoflow electrospray. The resulting data files were searched using an in-house MASCOT search engine. (MASCOT is a powerful search engine that uses mass spectrometry data to identify proteins from primary sequence databases [21].) Ion scores higher than 35 (*P *< 0.05) were considered significant. Only proteins matching at least two peptides in MASCOT were accepted (expectation: AZGP1). MASCOT position indicates (inversely) a relative abundance of the protein in secretome (the lower the score, the higher is the abundance). Matched sequences correspond to the number of nonredundant tryptic peptides mapped to the protein listed under the GenBank accession number. This indicates the confidence with which the protein was identified by mass spectrometry. NCBInr gi#, gene identification number from a comprehensive, nonidentical protein database maintained by National Center for Biotechnology Information. MRJ(L), mammalian relative of DnaJ long isoform.

### Determining changes in mRNA level of the key secreted proteins

We compared the expression levels of SPP1, AZGP1, VGF, SPARC, NPM1, and KiSS1 using quantitative RT-PCR to determine whether the change in the levels of secreted proteome was due to change in transcription. Real-time PCR comparison demonstrated reduced expression of SPP1 (17-fold), AZGP (10-fold), VGF (9.4-fold), SPARC (7-fold) and NPM1 (1.3-fold), and increased expression of KiSS1 (13-fold) in MDA-MB-435-MRJ(L) cells compared with the vector control (Figure 5b).

## Discussion

Members of the DnaJ/Hsp40 proteins are highly conserved and expressed in several tissues. They act as co-chaperones regulating protein folding, transport, translational initiation, and gene expression. The Hsp40 family of proteins is known to have co-chaperonic activity. Involvement of the Hsp40 family in tumorigenesis and malignant progression has yet to be completely elucidated. However, recent reports of the involvement of some Hsp40 members of distinct classes such as hTid I (class 3HDNAJA3) and HLJ1 (class DNAJB4) in modulation of tumor growth are emerging [26-32].

DNAJB6 (MRJ) isoforms belong to the Hsp40 superfamily, specifically class II. Our studies highlight the role of DNAJB6 isoform a, MRJ(L), as a negative regulator of tumor growth in breast cancer. We found that MRJ(L) expression is low in breast cancer cell lines. Constitutive expression of MRJ(L) in highly aggressive breast cancer cell lines MDA-MB-231 and MDA-MB-435 changes their *in vitro *and *in vivo *attributes of malignancy. Our analysis of *in vitro *attributes of tumor progression and metastasis revealed reduced wound healing, invasion, migration, and anchorage-independent growth. We observed reduced tumor growth rate and decreased lung colonization upon injection of MRJ(L) expressing cells in nude mice. These observations convincingly demonstrate a negative influence of MRJ(L) on tumor growth and metastasis.

The analysis of predicted domains of MRJ(L) revealed that MRJ(L) has a nuclear localization signal. Confocal microscopy studies using the EGFP fusion of MRJ(L) with wild-type or mutated NLS showed that a significant amount of MRJ(L) is localized to the nucleus, whereas the mutation in NLS rendered it cytoplasmic. The continued presence of the MRJ(L)-NLS-mut in the nucleus can be explained by the possibility that there exists an additional cryptic NLS that the PSORT II software may not have identified. Alternatively, MRJ(L) lacking the NLS may still interact with another nuclear protein and could be transported into the nucleus while attached to the other protein, as indicated in the recent report by Cheng and coworkers [22].

Secreted proteins play an important role in promoting tumor growth and interaction with the local microenvironment and facilitate its pathway to metastasis. MRJ(L) expressors of MDA-MB-435 showed reduced secretion of SPP1, SPARC, NPM1, VGF, and AZGP1, with concomitant increased expression of KiSS1. We found that MDA-MB-231 does not secrete VGF and AZGP1 or, as previously reported, SPP1 [24] and SPARC [25]. However, MRJ(L) expression in MDA-MB-231 leads to increased KiSS1 and decreased NPM1.

SPP1 is an RGD-binding glycoprotein that plays a prominent role in important steps in breast tumor growth and metastasis [18,33,34]. SPARC has also been shown to regulate adhesion and spreading of various types of tumor cells [35-38]. Antisense oligonucleotides directed against SPP1 and SPARC have been shown to inhibit proliferation and migration of MDA-MB-231 cells [39]. Also, SPARC has been reported to play a role in migration of breast cancer cells to bone [40]. However, there are conflicting reports about the precise role of SPARC in breast cancer. A study conducted by Koblinski and coworkers [25] indicated that SPARC expression inhibits MDA-MB-231 metastasis. However, the primary tumor growth rates (*in vivo *growth) of SPARC expressing MDA-MB-231 cells were not studied.

AZGP1 has been reported to be a potential quantitative marker of differentiation grade of oral tumors [41]. It is expressed by malignant prostatic epithelium and may serve as a potential serum marker for prostate cancer [42]. The levels of AZGP1 have been found to be higher in well differentiated than in moderate or poorly differentiated breast tumors [43]. Relevance of VGF in breast cancer has not yet been explored. However, peptide products of the neurotrophin-inducible gene VGF are produced in human neuroendocrine cells from early development and increase in hyperplasia and neoplasia [44].

NPM1 is a 37 kDa phosphoprotein with nucleo-cytoplasmic shuttling properties that is present at higher levels in proliferating cancer cells than in quiescent cells, and its increased expression in hepatocellular carcinoma correlates with clinicopathologic parameters [45]. Interestingly, NPM1 is not reported to be a secreted protein. Also, it lacks the traditional signal sequence. However, it is predicted with high confidence (by the Center for Biological Sequence Analysis SecretomeP^© ^2.0 Server [46]) to undergo a nonclassical protein secretion. Nonclassically secreted proteins should obtain an neural network output score (NN score) exceeding the normal threshold of 0.5; NPM1 has a score of 0.811.

The expression of KiSS1 (metastin) has been reported to exhibit an inverse correlation with human tumor progression and metastasis, and is either reduced or absent in various types of cancers [47-49]. Over-expression of KiSS1 in metastatic breast cancer cells or treatment of metastatic breast cancer cells with synthetic KiSS1 has been shown to reduce their metastatic potential [50,51]. Thus, the secreted proteome analysis revealed important molecular players downstream of MRJ(L), and the changes in the secreted proteome undoubtedly indicated a change from aggressive to nonaggressive phenotype. MRJ(L) expression upregulated KiSS1 and reduced the metastatic ability of the cells. It is important to note that we have sequenced the KiSS1 mRNA from the MRJ(L) expressors of MDA-MB-231 and MDA-MB-435 and found no mutation in the resident KiSS1 sequence (data not shown). Thus, the upregulated, resident KiSS1 protein is functional as a metastasis suppressor. Also, it is tempting to speculate that MRJ(L) may play a similar role in regulating melanoma metastasis by upregulation of KiSS1.

The changes in secreted protein levels could be due to several factors such as altered secretion rate, altered stabilization of proteins or altered transcription. The quantitative PCR analysis shows that the influence of MRJ(L) on the secreted proteins is at the transcriptional level, suggesting that MRJ(L) plays a role in mediating regulation of transcription of these genes. In a recent elegant study, Dai and colleagues [8] showed that the MRJ(S) and nuclear factor of activated T cells (NFAT)c3 could directly associate with one another in cardiomyocytes. Those investigators also found that MRJ(S) served as a potent inhibitor of NFAT transcriptional activity within the nucleus through a mechanism involving histone deacetylase recruitment in conjunction with heat shock. Heat shock is needed for the translocation of MRJ(S) from cytoplasm to the nucleus [8]. Thus, it is tempting to speculate that MRJ(L) is capable of similar transcription regulatory activities.

Tissue microarray analysis revealed that MRJ expression is lost as IDC grades advance. Importantly, total absence of MRJ staining in cases with lymph node metastasis was seen. The MRJ antibody does not distinguish between isoforms, and so we cannot comment on the presence or absence of each of the isoforms. Because of small sample size, it is premature to draw correlations between nuclear staining (which may correspond exclusively to MRJ [L]) and disease stage. However, it must be emphasized that none of the IDCs had positive nuclear staining (n = 24), whereas there was a higher number of positive nuclear staining in normal and benign (adenosis, simple hyperplasia, atypical hyperplasia) lesions (eight out of 17 stained). In fact, one of the ways in which a tumor can gain aggressive behavior is by spontaneous deletion or silencing of the MRJ gene, which will lead to absence of both isoforms. It is also likely that the expression of MRJ(S) is not ubiquitous in tissues, in contrast to the expression observed in cell lines, and this isoform may play similar or independent role(s) in regulating tumor growth.

## Conclusion

In summary, we found that MRJ(L) plays an important role in regulating breast cancer tumoriginicity and metastasis by altering transcription of key players in cancer progression and metastasis.

## Abbreviations

AZGP1 = zinc binding α_2_-glycoprotein 1; DMEM = Dulbecco's modified Eagle's medium; EGF = epidermal growth factor; EGFP = enhanced green fluorescent protein; GAPDH = glyceraldehyde 3-phosphate dehydrogenase; Hsp = heat shock protein; IDC = infiltrating ductal carcinoma; IRES = internal ribosome entry site; KiSS1 = melanoma metastasis suppressor; MRJ = mammalian relative of DnaJ; MRJ(L) = long isoform of MRJ; MRJ(S) = short isoform of MRJ; NFAT = nuclear factor of activated T cells; NLS = nuclear localization signal; NPM1 = nucleophosmin; PCR = polymerase chain reaction; RT = reverse transcription; SPARC = osteonectin; SPP1 = osteopontin; VGF = VGF nerve growth factor inducible.

## Competing interests

The authors declare that they have no current competing interests. However, RSS and LAS have a provisional patent filed on use of MRJ(L) in breast cancer prognosis and treatment.

## Authors' contributions

AM designed and conducted the experiments unless otherwise specified and wrote the manuscript. RF made various DNA constructs used. BM assisted AM in carrying out the experiments. MR and LP performed secreted proteome analysis by tandem mass spectrometry. YX and JJ performed the quantitative RT-PCRs, JK verified the tissue microarray results, and LS assisted in the nude mice study and secreted proteome studies. RS participated in designing the experiments and writing the manuscript, and carried out nude mice studies. All authors read and approved the final manuscript.
